# Do long delay conditioned stimuli develop inhibitory properties?

**DOI:** 10.3389/fpsyg.2015.01606

**Published:** 2015-10-23

**Authors:** Martha Escobar, W. T. Suits, Elizabeth J. Rahn, Francisco Arcediano

**Affiliations:** ^1^Department of Psychology, Auburn UniversityAuburnm, AL, USA; ^2^Department of Psychology, Oakland UniversityRochester, MI, USA; ^3^Department of Psychology, Seminole State CollegeSanford, FL, USA; ^4^Department of Neurobiology, Evelyn F. McKnight Brain Institute, University of Alabama at BirminghamBirmingham, AL, USA

**Keywords:** inhibition, inhibition of delay, long-delay conditioning, timing, conditioned inhibition, latent inhibition

## Abstract

In long-delay conditioning, a long conditioned stimulus (CS) is paired in its final segments with an unconditioned stimulus. With sufficient training, this procedure usually results in conditioned responding being delayed until the final segment of the CS, a pattern of responding known as inhibition of delay. However, there have been no systematic investigations of the associative structure of long delay conditioning, and whether the initial segment of a long delay CS actually becomes inhibitory is debatable. In an appetitive preparation with rat subjects, the initial segment of long delay CS A passed a retardation (Experiment 1a) but not a summation (Experiment 1b) test for conditioned inhibition. Furthermore, retardation was observed only if long delay conditioning and retardation training occurred in the same context (Experiment 2). Thus, the initial segment of a long delay CS appears to share more characteristics with a latent inhibitor than a conditioned inhibitor. Componential theories of conditioning appear best suited to account for these results.

## Introduction

In general terms, a conditioned stimulus (CS) can have one of three relationships to the unconditioned stimulus (US): The CS may provide no information about the US occurrence (neutrality), it may provide information about US delivery (excitation), or it may provide information about US omission (inhibition). Labeling a stimulus as a ‘CS’ suggests that the stimulus as a whole becomes conditioned (i.e., produces a CR). However, there seem to be instances in which the stimulus as a whole does not elicit a CR. For example, long stimuli trained to signal US delivery in their final segment (so-called long delay conditioning training) usually elicit little conditioned responding during their initial segment. This attenuated responding is observed even if they elicit robust conditioned responding during their final segment. That is, unlike other forms of conditioning, in long delay conditioning the latency of the CR increases rather than decreases as training progresses (but see, e.g., Schneiderman and Gormezano, 1964; Gormezano, 1972). Pavlov (1927) coined the term *inhibition of delay* to describe this pattern of behavior and to reflect his assumption that inhibitory processes operated during long delay conditioning and resulted in delaying of the conditioned response until the moment in which the US occurred. This inhibitory process was disrupted if a novel stimulus was presented simultaneously with the long delay CS, which resulted in an immediate elicitation of the conditioned response (i.e., disinhibition). Supporting Pavlov’s assumptions, Rescorla (1967) provided evidence of inhibition to the initial segment of a long delay CS using a summation test in a fear conditioning preparation. In his preparation, the initial segment of the CS attenuated fear of the highly excitatory experimental context. Unfortunately, both Pavlov’s (1927) and Rescorla’s (1967) results can be explained without invoking the construct of inhibition. For example, Pavlov’s observation of disinhibition could simply reflect generalization decrement stemming from presentation of a novel stimulus together with the long delay CS. Similarly, Rescorla’s observation of summation may be due to non-specific disruption of responding to the context (i.e., external inhibition; cf. Pavlov, 1927), or generalization decrement of responding to the context due to the addition of the long-delay CS.

A potential way to circumvent the problems identified with Pavlov’s (1927) and Rescorla’s (1967) identification of the initial segment of a long delay CS as inhibitory is to implement the so-called *two-test strategy* (cf. Rescorla, 1969), which is based on using both summation and retardation tests to determine whether a stimulus is inhibitory. In a summation test, a target (putative inhibitor) is presented in compound with a known excitor, and evidence of inhibition is defined as attenuation of responding to the excitor as compared to a condition in which the target is absent. But Rescorla (1969) suggested that this test alone was not sufficient because processes other than inhibition could explain the observed response attenuation. For example, it is possible that inhibition training results in enhanced attention to the target and, at test, the presence of the inhibitor shifts attention away from the excitor, thereby attenuating responding to it. Rescorla (1969) proposed the concurrent use of a *retardation test*, in which the target (putative inhibitor) is paired with the US, and evidence of inhibition is defined as retarded acquisition of excitatory conditioned responding as compared to a condition in which no inhibitory training took place. Rescorla (1969) suggested that this test alone was insufficient to demonstrate inhibition because other mechanisms can also produce this pattern of responding. For example, if inhibition training resulted in attenuated attention to the target, its learning rate would decrease resulting in retardation. Because the alternative explanations for summation and retardation tests are mutually exclusive, Rescorla (1969) proposed that both tests are needed to determine that a stimulus has inhibitory behavioral control. Thus, if the initial segment of a long delay CS is inhibitory, it should attenuate responding to an excitor presented in compound with it *and* exhibit retardation of acquisition of excitatory behavioral control if paired with US delivery. That is, to be considered inhibitory, the initial segment of a long delay CS should pass both summation and retardation tests for conditioned inhibition.

An alternative to the inhibition view is that the initial segment of the long delay stimulus comes to signal something other than omission of an expected US. For example, the initial segment of the stimulus may simply remain neutral and acquire neither excitatory nor inhibitory behavioral control. If this was the case, the initial segment of the stimulus should readily develop excitatory behavioral control if reinforced, and should not attenuate responding to an excitor presented in compound with it. A third alternative is that the initial segment of the CS may exhibit attenuated response potential as a consequence of either a decrease in associability of the initial segment of the CS or the development of strong associations to the context, both of which have been proposed as mechanisms underlying latent inhibition (see Escobar et al., 2002; Escobar and Miller, 2010, for a review). In this case, the initial segment of the CS should exhibit retardation of acquisition of excitatory behavioral control if paired with US delivery, and should not attenuate responding to a conditioned excitor presented in compound with it (Rescorla, 1971). That is, if the initial segment of a long delay CS was latently inhibited, it should pass a retardation but not a summation test for conditioned inhibition. The present studies were designed to contrast these three alternatives.

Rats received long delay conditioning with a 60-s CS (CS A) that signaled delivery of sucrose pellets 55 s after CS onset. Conditioned responding was assessed in terms of number of head entries (nose pokes) into a niche containing the feeding cup. This procedure resulted in animals’ producing few nose poking responses during the initial segment of CS A, and a gradually increasing number of responses as the CS presentation progressed, with a response peak at about the time of US expectation (i.e., inhibition of delay). In Experiment 1a, the initial segment of the CS was then paired with the US (delivered 10 s after CS onset) to assess retardation of acquisition of the nose poking conditioned response. Experiment 1b assessed whether the initial segment of the long delay CS could attenuate responding to an independently trained, discrete excitor (i.e., a summation test). Experiment 2 assessed the possibility that the initial segment of the long delay CS had developed into a latent inhibitor rather than a conditioned inhibitor.

Notably, we selected an appetitively- rather than an aversively motivated preparation for the present studies. Conditioned inhibition is often studied in the framework of fear conditioning, although there are a few demonstrations of inhibition in appetitive preparations (e.g., Nelson, 2002; Williams et al., 2008a). An appetitive preparation was chosen because appetitive conditioning better allows for online measuring of the development of temporally specific behavior; thus, we could readily measure the development of inhibition and excitation across the duration of the CS.

## Experiments 1A And 1B

Experiments 1a and b investigated whether the initial segment of a long delay CS becomes inhibitory by using retardation and summation tests. Experiment 1a assessed retardation in the development of excitatory response potential to the initial segment of the long delay CS. Experiment 1b tested whether the initial segment of the long delay CS attenuated responding to a transfer excitor trained to predict US delivery during its initial segment. If retarded acquisition of a response to the initial segment of the long delay CS was observed in Experiment 1a, the possibility that it is neutral can be rejected. Furthermore, if attenuation of responding to the initial segment of the transfer excitor was also observed in Experiment 1b, the view that long delay conditioning results in the development of inhibition to this stimulus segment would be supported. However, if retardation was observed in Experiment 1a but summation was not observed in Experiment 1b, the assumption that the initial segment of the long delay CS becomes inhibitory would not be supported and an alternative mechanism would be suggested. As a possibility, the response potential of the initial segment of the CS may be attenuated due to a process akin to latent inhibition, which should result in a retarded acquisition of responding to the initial segment of the stimulus and no attenuation of responding to the transfer excitor (Rescorla, 1971).

**FIGURE 1:**
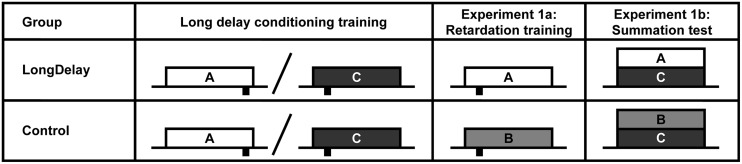
**Design of Experiments 1a and b.** The white, light gray, and dark gray rectangles represent CSs A, B, and C, respectively. CSs A and B were a 60-s white noise or a 60-s flashing light, counterbalanced within groups. CS C was a 60-s 2,900-Hz tone. Sucrose pellet USs (represented by black rectangles under the time line) were delivered at either 55 or 10 s after CS onset (represented at the appropriate location under the time). Onset of CSs A and C (Group LongDelay) and B and C (Group Control) during summation testing (Experiment 1b) were simultaneous. Slashes represent intermixed presentation of CSs during training. See text for details.

In both Experiments 1a and b, CS A was trained as a long delay predictor of the US. Thus, the US (sucrose pellets) was delivered 55 s after onset of the 60-s CS. The US was delivered at 55 s to ensure that subjects used CS duration rather than CS termination cues to determine time of delivery of the US. A second CS, C, was trained as a short delay predictor of the US with US delivery 10 s after onset of the 60-s CS (see **Figure 1**). Training with the short delay CS had two purposes: first, it allowed for a measure of response discrimination (responses were expected to peak during the initial segment of CS C and the final segment of CS A); second, the initial segment of CS C could be used as a transfer excitor for summation testing (for consistency, the C-US trials were included in all studies of the series). Because the development of inhibition appears to be highly dependent on the number of non-reinforced training presentations of the putative inhibitor (e.g., Yin et al., 1994; Stout et al., 2004), nine times more CS A than CS C trials were delivered, for a total of 450 and 50 trials, respectively. After training was completed, conditioned inhibition to the initial segment of CS A was assessed.

In Experiment 1a, after completion of the long delay conditioning training phase, subjects began retardation training (see **Figure 1**). During these trials, CS A, which had been trained to predict US delivery 55 s after CS onset, predicted US delivery 10 s after onset (LongDelay group). In the Control condition, subjects received equivalent training with a novel CS, B. Thus, development of conditioned responding to the initial segment of the putative inhibitor, CS A, was compared to development of conditioned responding to the equivalent segment of a neutral stimulus. Experiment 1b assessed inhibition using a summation test. In this test, following completion of the long delay conditioning training phase, subjects in the Long Delay condition received presentations of the compound of CSs A and C. CS C predicted US delivery 10 s after onset; thus, if the initial segment of CS A was inhibitory, it should attenuate responding to the initial segment of CS C. In the Control condition, subjects were presented with the compound of CS C and novel CS B. Because B had received no previous training, it was not expected to affect responding to CS C beyond external inhibition; thus, if inhibition had developed, responding should be attenuated in the initial segment of the AC compound more than in the initial segment of the BC compound.

### Materials and Methods

#### Subjects

The subjects were 48 male (227–264 g in Experiment 1a, 219–257 g in Experiment 1b) albino rats (Holtzman stock, Harlan Labs). The 24 subjects in each experiment were randomly assigned to one of two groups, LongDelay or Control (*n*s = 12). Subjects were housed in pairs in standard plastic cages with wire lids in a vivarium maintained on a 12-hr light/12-hr dark cycle. All experimental manipulations occurred during the light portion of the cycle. Water was available *ad lib* to all subjects. A food deprivation schedule was imposed during the week preceding the initiation of the experiment such that feedings were gradually reduced to maintain animals at approximately 85% of their free-feeding weight. Food (regular rat chow) was provided approximately 1 h after completion of each experimental session. From the time of arrival to the laboratory until initiation of the study, animals were handled for 30 s every other day. All cagemates were assigned to different groups. The research was conducted in accordance with the “Principles of laboratory animal care” (NIH publication No. 86-23, revised 1985) and all procedures were approved by the Auburn University Institutional Animal Care and Use Committee.

#### Apparatus

The apparatus consisted of eight Med Associates standard rat chambers (30.5 cm long × 24.1 cm wide × 21.0 cm high). The sidewalls of each chamber were made of aluminum sheet metal, and the front wall, back wall, and ceiling of the chamber were made of transparent polycarbonate. The floor was constructed of 4.8-mm stainless steel rods, spaced 1.6 cm center-to-center. Each chamber was housed in a melamine sound attenuation cubicle equipped with an exhaust fan that provided a constant, 70 dB background noise (this and all subsequent sound pressure level measures were performed using the A scale). All chambers were equipped with a pellet dispenser that could deliver 45-mg sucrose pellets into a cup located inside a niche (5.1 cm long × 5.1 cm wide × 5.1 cm high). The niche was placed on a side wall, 1.5 cm above the grid floor, and was equipped with an infrared photo beam, which when disrupted could be used to detect the number of head entries into the niche; this was used as our dependent variable. All chambers were also equipped with a speaker mounted above the pellet dispenser and two speakers mounted on the opposite wall. These speakers could produce a 2,900-Hz tone or a 4,500-Hz tone, and a white noise or an 800-Hz tone, respectively. All auditory stimuli were delivered at an intensity of 10 dB above background. A 1.12-Watt (#1820) flashing houselight (0.50 s on/0.50 s off) was used as a visual stimulus. All stimulus events were programmed and all data was recorded using MedPC software.

#### Procedure

**Figure 1** presents the critical aspects of Experiments 1a and b. CSs A and B were the white noise and the flashing houselight, counterbalanced within groups. CS C was the 2,900-Hz tone. When delivered, CSs A, B, and C were 60 s in duration. The US consisted of the delivery of two 45-mg sucrose pellets 55 s after CS A onset and 10 s after CS C onset. All sessions were 120 min in duration, except for the test session, which was 60 min in duration. Throughout training and testing, the chamber was dark, with the exception of presentations of the houselight as a stimulus. The procedure was identical for both studies, with exception of the treatment provided on Day 27 (retardation training in Experiment 1a, summation testing in Experiment 1b), which is detailed below. Note that counterbalancing the noise and houselight as CSs A and B and using the tone as CS C resulted in some animals receiving summation testing with an auditory–auditory compound and other animals being tested with an auditory-visual compound. Pilot research in our laboratory suggested that the noise and houselight acquire equivalent control of nose poke behavior, and no differences in responding based on stimulus nature were observed in any of the present studies.

##### Acclimation

On Day 1, all subjects were acclimated to the experimental context and to retrieving pellets from the niche. Sucrose pellets were delivered on a fixed-time 5-min schedule. During this session subjects were exposed to two presentations of all stimuli in the order houselight, tone, noise, houselight, noise, tone, with a mean intertrial interval of 15 min in order to reduce unconditioned fear to these stimuli.

##### Long delay conditioning training

On Days 2–26, all subjects received 18 daily CS A-US pairings (US delivery occurred 55 s after onset of CS A) as well as 2 daily CS C-US pairings (US delivery occurred 10 s after onset of CS C). Thus, A was trained as a long delay CS and C was trained as a short delay CS. Although the C-US pairings were irrelevant to the retardation studies, they were included in all studies of the series for consistency of treatment with the summation study. Two schedules of training were used on alternate days. Trials 3 and 14 (Schedule 1) or 7 and 18 (Schedule 2) were designated as C trials. In both schedules, the mean intertrial interval was 6 (±3) min. Probe trials (presentation of the CS without US delivery) were included to test for acquisition of the response without contamination from US presentation. The 90, 180, 270, 360, and 450th presentation of A and the 10, 20, 30, 40, and 50th presentation of C were designated as probe trials.

##### Experiment 1a: retardation training

On Day 27, subjects received either 20 CS A (Group LongDelay) or 20 CS B (Group Control) presentations, with a mean intertrial interval of 6 (±3) min. During 19 of the A-US and B-US trials, US delivery occurred 10 s after onset of the 60-s stimuli; thus, conditioned responding was expected to develop to the initial segment of the CS. The 20th trial was designated as a probe trial (i.e., it was not reinforced).

##### Experiment 1b: summation test

On Day 27, subjects received two presentations of either AC (Group LongDelay) or BC (Group Control), with an intertrial interval of 15 min. All stimuli were 60 s in duration and no stimulus presentation was followed by delivery of the US.

#### Data Analysis

Number of head entries during all training and test sessions was recorded in 5-s bins. For purposes of analysis, the three first and three last bins (i.e., the first and last 15 s) of the 60-s CS were used as a measure of responding during the initial and final segments of the CS. The occurrence of long delay conditioning was assessed by analyzing the last probe trial, retardation (Experiment 1a) was assessed by analyzing the retardation training probe trial, and summation was assessed by analyzing the average responding to the two test trials. Outlier scores for each statistical analysis were excluded using Grubbs’ test (e.g., Grubbs and Beck, 1972), with the constraint that a maximum of one data point would be excluded from any analyzed segment (i.e., the test was performed with no iterations). In Experiment 1a, application of the outlier criterion resulted in the data from one subject in Group LongDelay being excluded from the long delay conditioning analysis, and the data from one subject in Group LongDelay being excluded from the retardation test analyses. In Experiment 1b, the data from two subjects in Group LongDelay were excluded from the long delay conditioning analysis, and the data from one subject in Group Control were excluded from the summation test analyses.

### Results and Discussion

#### Experiment 1a

**FIGURE 2:**
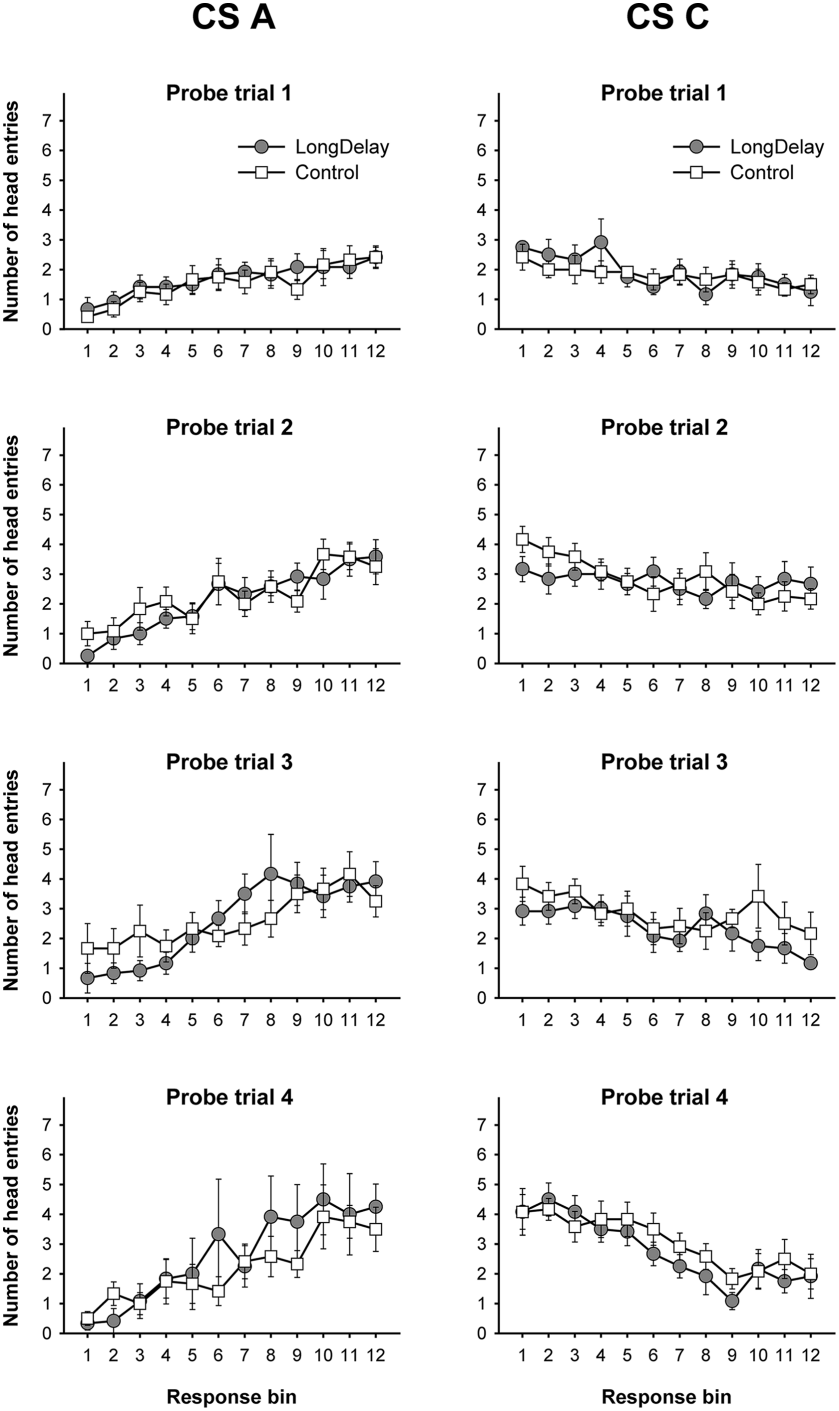
**Long delay conditioning, Experiment 1a.** Responding to CS A **(left)** and CS C **(right)** during each of the 12 5-s bins of the first four probe trials of long delay conditioning training. US delivery was expected at 55 s (Bin 11) or 10 s (Bin 2) after onset of CSs A and C, respectively. Probe trials for CS A occurred every 90th trial, whereas for CS C they occurred every 10th trial. Brackets represent the standard error of the means.

**FIGURE 3:**
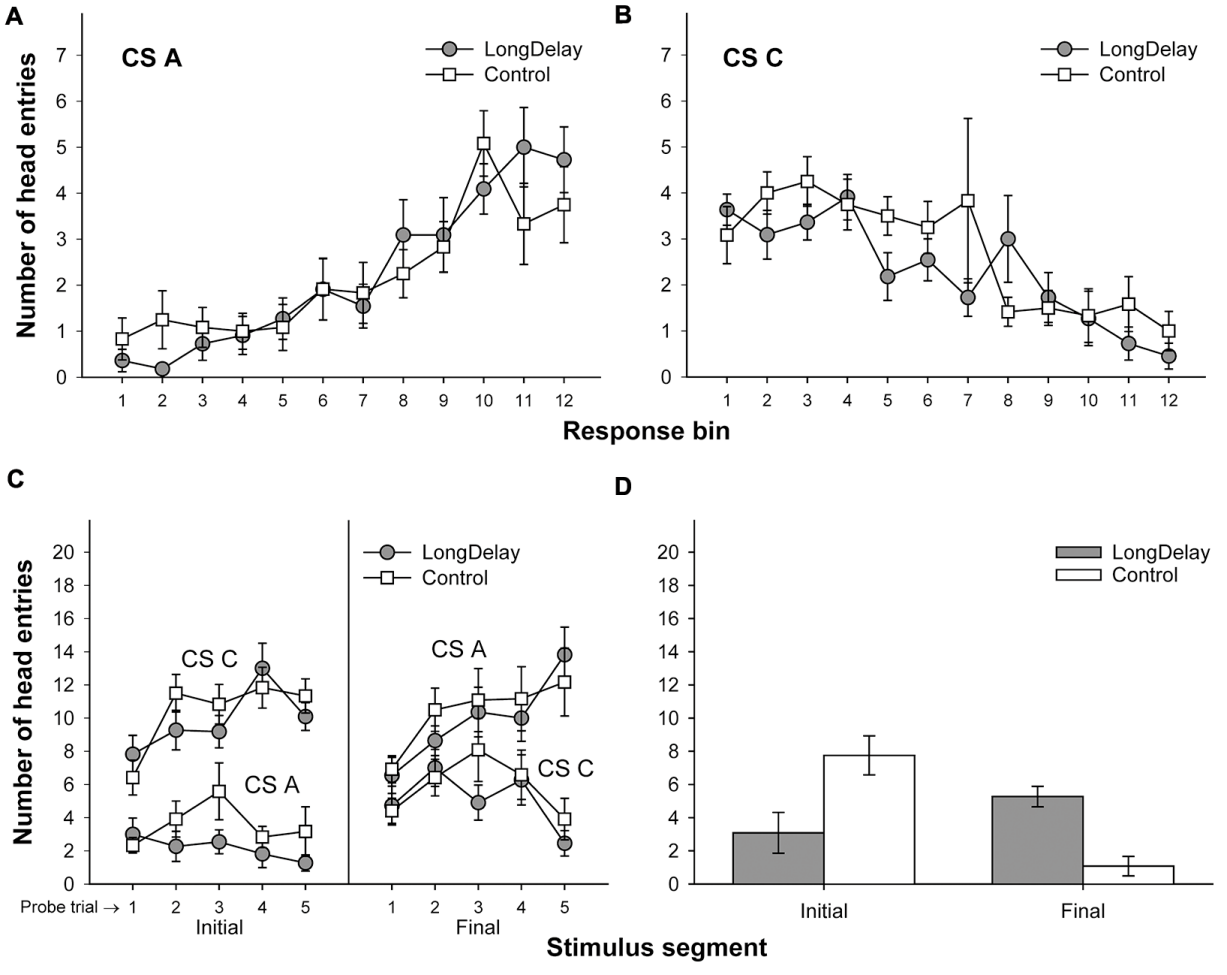
**Results of Experiment 1a.** The top panels represent responding to CSs A **(A)** and C **(B)** during each of the 12 5-s bins of the last probe trial of long delay conditioning training. US delivery was expected at 55 s (Bin 11) or 10 s (Bin 2) after onset of CSs A and C, respectively. **(C)** Presents responding to the initial (average of Bins 1–3) and final (average of Bins 10–12) segments of CSs A and C during the five probe trials of long delay conditioning training. **(D)** Presents responding to the initial and final segments of CSs A (Group LongDelay) and B (Group Control) during the probe trial of retardation training. Brackets represent the standard error of the means.

Long delay conditioning training occurred as expected, with subjects in both groups exhibiting more responding to the final than the initial segment of CS A and more responding to the initial than the final segment of CS C (see **Figures 2** and **3**). The data of greatest interest were obtained during the retardation training phase. Acquisition of conditioned responding to the initial segment of long delay CS A was slower than acquisition of conditioned responding to the equivalent segment of novel CS B. That is, the initial segment of the long delay CS exhibited retarded acquisition of excitatory behavioral control. The following analyses support these conclusions.

##### Long delay conditioning training

Responding to long delay CS A and short delay CS C acquired temporal-specific properties as training progressed. **Figure 2** presents the bin-by-bin data collected during first four probe trials with both stimuli (equivalent data for the last probe trial of the long delay conditioning phase is presented in **Figures 3A,B** described below). The figure evidences that responding initially occurred throughout the duration of both CSs A and C, and as training progressed became constrained to the segment of the stimulus more contiguous with reinforcement. Acquisition occurred in a similar fashion in all subsequent experiments. To ensure that responding to the long and short delay CSs had reached equivalent levels at the end of training across groups, a 2 (group, between groups factor) × 2 (stimulus: A vs. C, within-subjects factor) × 2 (segment: initial vs. final, within-subjects factor) analysis of variance (ANOVA) was conducted on the last probe trial administered during the long delay conditioning training phase. This analysis revealed a main effect of segment, *F*(1,21) = 4.63, *MSE* = 13.07, *p* < 0.05, as well as an interaction of Stimulus × Segment, *F*(1,21) = 87.42, *MSE* = 21.98, *p* < 0.0001 (see **Figures 3A–C**). No other main effect or interaction was significant, all *p*s > 0.28. *Post hoc* comparisons using the Bonferroni correction revealed that subjects in both groups exhibited more responding to the final segment of CS A than the equivalent segment of CS C, and more responding to the initial segment of CS C than the equivalent segment of CS A, all *p*s < 0.0005 (see **Figures 3A–C**).

##### Retardation test

A 2 (group) × 2 (segment) ANOVA was performed on the number of head entries recorded during the test stimulus probe presentation. This analysis yielded a main effect of segment and a Group × Segment interaction, *F*s(1,21) = 5.71 and 22.24, *MSE* = 10.10, *p*s < 0.05 and 0.0005, respectively. The main effect of group was not significant, *F* < 1. A series of pair-wise comparisons derived from the 2 × 2 ANOVA were conducted to analyze responding in the initial and final segments of the test stimulus. Groups LongDelay and Control differed in level of responding to the initial segment of the test stimulus, *F*(1,21) = 7.49, *MSE* = 16.63, *p* < 0.05, reflecting retarded acquisition of conditioned responding in Group LongDelay. Groups LongDelay and Control also differed in level of responding to the final segment of the test stimulus, *F*(1,21) = 24.27, *MSE* = 4.15, *p* < 0.0001; that is, Group LongDelay continued to exhibit more responding to the final segment of CS A than Group Control (**Figure 3D**). A further analysis included responding during Bins 1 and 2 of all CS A retardation trials, which represent conditioned responding prior to presentation of the US (Bin 3, which was included for the probe trial analyses, occurred after US presentation and thus represents both conditioned and unconditioned responding). A 2(group) × 5(block of 4 trials) ANOVA revealed main effects of group, *F*(1,21) = 8.44, *MSE* = 53.12, *p* < 0.01, and block, *F*(4,84) = 5.49, *MSE* = 2.97, *p* < 0.001. The interaction was borderline significant, *F*(4,84) = 2.43, *p* = 0.05. Responding in the two groups did not differ in the first block, *p* > 0.20, but differed in all subsequent blocks, *p*s < 0.05, suggesting that behavioral control by CS A was equivalent in the two groups at the initiation of retardation training, and retardation emerged as training progressed.

#### Experiment 1b

**FIGURE 4:**
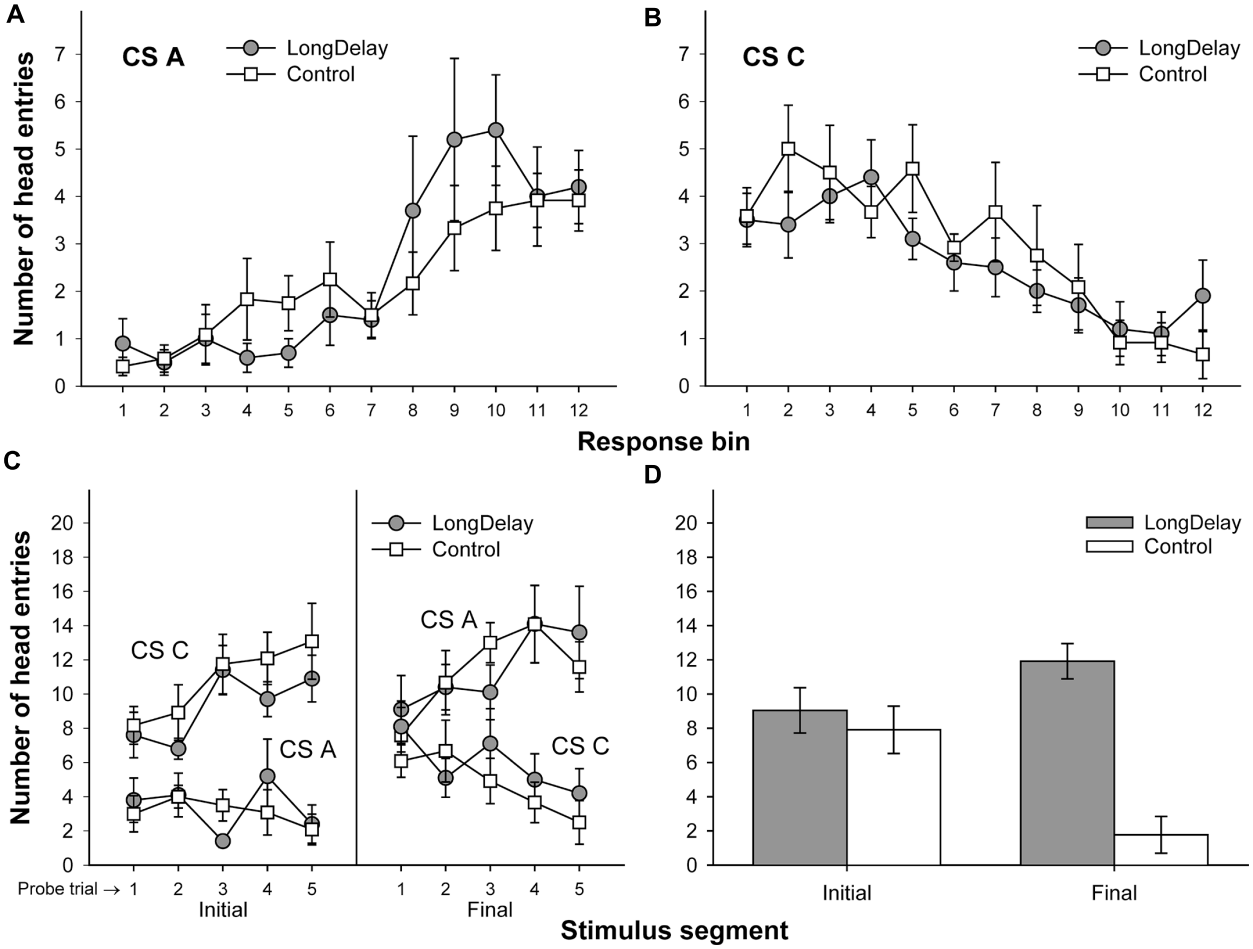
**Results of Experiment 1b.** The top panels represent responding to CSs A **(A)** and C **(B)** during each of the 12 5-s bins of the last probe trial of long delay conditioning training. US delivery was expected at 55 s (Bin 11) or 10 s (Bin 2) after onset of CSs A and C, respectively. **(C)** Presents responding to the initial (average of Bins 1–3) and final (average of Bins 10–12) segments of CSs A and C during the five probe trials of long delay conditioning training. **(D)** Presents responding to the initial and final segments of test compounds AC (Group LongDelay) and BC (Group Control) during the two summation test trials (averaged). Brackets represent the standard error of the means.

Long delay conditioning resulted in subjects in both groups exhibiting more responding to the initial than the final segment of CS C and more responding to the final than the initial segment of CS A (see **Figure 4**). However, low responding to the initial segment of CS A was not indicative of inhibition, as assessed with a summation test. CS A failed to attenuate responding to the initial segment of CS C beyond any attenuation produced by a stimulus that did not receive long delay conditioning training, CS B. These conclusions are supported by the following statistical analyses.

##### Long delay conditioning training

A 2 (group, between groups factor) × 2 (stimulus: A vs. C, within-subjects factor) × 2 (segment: initial vs. final, within-subjects factor) ANOVA conducted on the last probe trial administered during long delay conditioning training revealed an interaction of Stimulus × Segment, *F*(1,20) = 58.25, *MSE* = 33.77, *p* < 0.0001. No other main effect or interaction was significant, all *p*s > 0.13. *Post hoc* comparisons using the Bonferroni correction revealed that subjects in both groups exhibited more responding to the final segment of CS A than the equivalent segment of CS C, and more responding to the initial segment of CS C than the equivalent segment of CS A, all *p*s < 0.05 (see **Figures 4A–C**).

##### Summation test

A 2 (group) × 2 (segment) ANOVA was conducted on the mean of the data collected during the two test compound presentations to assess the occurrence of conditioned inhibition. This analysis yielded a main effect of group, *F*(1,21) = 19.11, *MSE* = 19.09, *p* < 0.0005, and a Group × Segment interaction, *F*(1,21) = 15.74, *MSE* = 14.80, *p* < 0.001. Planned comparisons derived from the 2 × 2 ANOVA revealed no differences between groups during the initial segment of the test compound presentation, *F* < 1. Indeed, there was an ordinal difference between groups in the direction *opposite* to inhibition; that is, the LongDelay group responded (non-significantly) more than the Control group to the test compound (see the bottom-right panel of **Figure 4**). This difference between groups most likely reflects generalization decrement affecting the Control group more than the LongDelay group. Indeed, in the Control group, responding based on Stimulus C dropped from a mean (±SEM) of 12.36 (±2.14) responses to a mean of 7.91 (±1.08) responses. One could argue that this large generalization decrement in the Control group made it difficult to detect inhibition in the LongDelay group, and a more appropriate control condition would have included a comparison between responding to the AC compound and responding to Stimulus C alone. However, decrements in responding during a summation test may come from three sources: external inhibition produced by the added stimulus, generalization decrement due to the change from the training stimulus configuration to the test stimulus configuration, and conditioned inhibition *per se*. Adding a novel stimulus to the transfer excitor (Stimulus C) controls for decrements due to external inhibition and generalization decrement; thus, to be considered inhibitory, the putative inhibitor should attenuate responding to C beyond the attenuation produced by these factors. Furthermore, a comparison of responding to the initial segment of Stimulus C during the last probe trial and responding to the initial segment of the test compounds (AC and BC) revealed a main effect of stimulus (C vs. test compound), *F*(1,22) = 14.80, *MSE* = 14.22, *p* < 0.001, but neither a main effect of Group nor a Group × Stimulus interaction, *F*s < 1. Thus, generalization decrement occurred in both groups and the failure to detect any response attenuation beyond generalization decrement suggests a failure to obtain evidence of inhibition with the summation test.

A possible concern with the present study was that half of the subjects were tested on the compound of two auditory stimuli and, the remaining half, on the compound of one auditory and one visual stimulus. The ANOVA was repeated including the nature of stimulus A (visual vs. auditory) as a factor. This analysis replicated the results of the previous analysis, yielding a main effect of group and a Group × Segment interaction, *F*s(1,18) > 10.02, *p*s < 0.01, but neither a main effect of stimulus nor an interaction of stimulus with any of the other factors, *F*s < 1.35, *p*s > 0.26.

A comparison of responding to the final segment of the test compounds revealed more head entries during presentation of AC (Group LongDelay) than BC (Group Control), *F*(1,21) = 46.17, *MSE* = 12.79, *p* < 0.0001. This difference was expected because CS A predicted US delivery 55 s after CS onset. In contrast, an expectation of US delivery during the second half of the BC compound presentation was unlikely because neither B nor C predicted US delivery during this period. Thus, presentation of the AC compound seemingly resulted in responding based on the expected time of US delivery provided by both CS C (initial segment) and CS A.

#### Conclusion

Long delay conditioning training established CS A as a predictor of the US in its final segment. Consequently, subjects exhibited little responding to the initial segment of CS A, which constitutes the typical inhibition of delay response pattern (**Figures 2**, **3A,B** and **4A,B**). When inhibition was assessed, the initial segment of CS A was slower than the initial segment of a novel stimulus in acquiring control of conditioned responding; that is, the initial segment of CS A passed a retardation test for conditioned inhibition (Experiment 1a). However, the initial segment of CS A did not pass a summation test (Experiment 1b). Thus, Experiment 2 focused on alternative perspectives that use mechanisms other than conditioned inhibition to explain the response pattern that characterizes long delay conditioning.

## Experiment 2

Experiment 1a detected retardation of acquisition of a conditioned response to the initial segment of a CS that had undergone long delay conditioning training. That is, the stimulus passed a retardation test for conditioned inhibition. This same training, however, did not result in the initial segment of the long delay CS attenuating responding to the initial segment of an excitor trained to predict the US 10 s after CS onset (Experiment 1b). That is, the initial segment of the long delay CS failed to pass a summation test for conditioned inhibition. Although one could argue that passing both summation and retardation tests for conditioned inhibition is not a necessary requirement to consider a stimulus inhibitory (e.g., Williams et al., 1992; Papini and Bitterman, 1993; see the Discussion for elaboration), one should wonder whether the initial segment of the CS indeed became a ‘true’ conditioned inhibitor.

As an alternative to conditioned inhibition, consider the possibility that latent inhibition developed to the initial segment of the long delay CS. In a latent inhibition procedure (cf. Lubow and Moore, 1959), a stimulus that is repeatedly presented in the absence of a US is later retarded in acquiring or expressing an association with the US. Notably, latent inhibition has long been regarded as distinct from conditioned inhibition because latent inhibitors do not pass a summation test for conditioned inhibition (i.e., they fail to attenuate responding to a known excitor; Rescorla, 1967) and CS preexposure results not only in retardation of excitatory responding, but also in retardation of inhibitory responding (e.g., Baker and Mackintosh, 1977; Friedman et al., 1998). Experiments 1a and b provide some support for this hypothesis because, as is the case with latent inhibitors, the initial segment of the CS passed a retardation but not a summation test. Furthermore, one of the defining characteristics of latent inhibition is that it is highly context-specific. That is, when preexposure treatment (i.e., CS alone presentations) is given in Context 1 and conditioning treatment (i.e., CS-US pairings) is given in Context 2, latent inhibition is greatly attenuated (e.g., Channell and Hall, 1983; Hall and Minor, 1984; Rosas and Bouton, 1997). Indeed, the context-specificity of latent inhibition has served to differentiate among different theoretical approaches to the phenomenon (for a review, see Escobar and Miller, 2010). Applied to the present studies, if retardation of acquisition following our long delay conditioning training resulted from preexposure to the initial segment of the CS, then changing the context between long delay conditioning training and retardation training should greatly attenuate the retardation effect.

**FIGURE 5:**
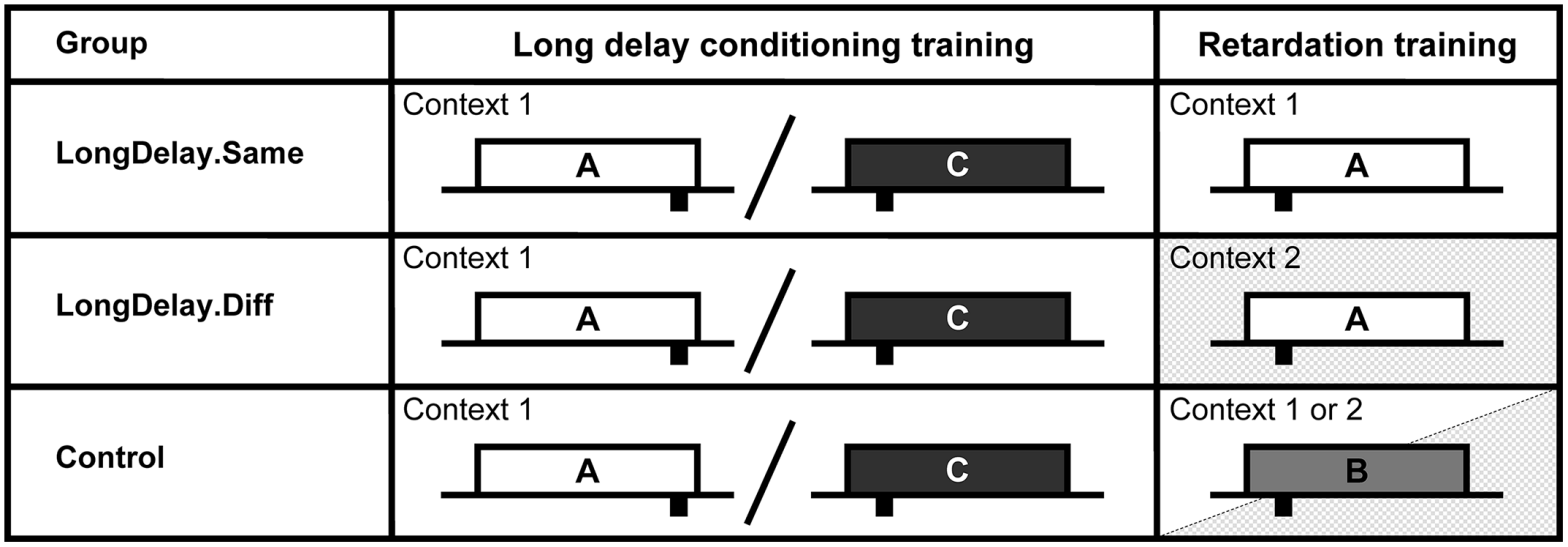
**Design of Experiment 2.** The white, light gray, and dark gray rectangles represent CSs A, B, and C, respectively. CSs A and B were a 60-s white noise or a 60-s flashing light, counterbalanced within groups. CS C was a 60-s 800-Hz tone. Sucrose pellet USs (represented by black rectangles under the time line) were delivered at either 55 or 10 s after CS onset (represented at the appropriate location under the time). Long delay conditioning training for all groups occurred in Context 1 (white background). Retardation training occurred in Context 1 for Group LongDelay.Same, Context 2 (shaded background) for Group LongDelay.Diff, and in either Context 1 or Context 2 for Group Control. Slashes represent intermixed presentation of CSs during training. See text for details.

In Experiment 2, three groups of rats received long delay conditioning training of CS A in Context 1. Then, all groups received retardation training such that the initial segment of a 60-s CS came to signal US delivery 10 s after onset. In the Long Delay condition, these retardation training trials involved pairings of CS A and the US, whereas in the Control condition, they involved pairings of CS B and the US. Retardation training occurred either in the same or a different context from that of long delay conditioning. Group LongDelay.Same received CS A-US pairings in Context 1, Group LongDelay.Diff received CS A-US pairings in Context 2, and Group Control received CS B-US pairings in either Context 1 or Context 2 (CS B was a novel stimulus used to provide a baseline of acquisition; see **Figure 5**). Based on the results of Experiment 1a, retardation of acquisition was expected in Group LongDelay.Same relative to Group Control. Furthermore, if long delay conditioning results in latent inhibition of the initial segment of the long delay CS, no evidence of retardation would be expected in Group LongDelay.Diff relative to Group Control.

### Materials and Methods

#### Subjects and Apparatus

The subjects were 32 male (355–546 g) albino rats acquired, housed, and maintained as in the previous studies. All animals had previously served as subjects in a study using a different set of stimuli and an aversively motivated task. Eleven subjects were assigned to each the LongDelay.Same and LongDelay.Diff groups, and the remaining 10 subjects were assigned to the Control group. The apparatus were the same as those described for the previous studies.

#### Procedure and Data Analysis

**Figure 5** presents the critical aspects of Experiment 2. The procedure was the same as that described for Experiment 1a, with the following exceptions. Two contexts (the grid and plexi enclosures) were used in this study. The *grid enclosure* was the chamber as described in Experiment 1a. The *plexi enclosure* used the same chambers as the grid enclosure, but a smooth, transparent, Plexiglas sheet was used to cover the grids and a pattern of alternating black and white stripes was used to cover the clear walls of the enclosure. For each animal, different physical chambers were used as enclosures grid and plexi. Designations of enclosures grid and plexi as Contexts 1 and 2 was counterbalanced within groups. The flashing light and noise served as CSs A and B, counterbalanced within groups. The 800-Hz tone served as CS C for all subjects.

On Day 1, all subjects were acclimated to the experimental context as described for Experiments 1a and b, except that they received a 60-min exposure to each of Contexts 1 and 2. On Days 2–26, all subjects received long delay conditioning training as described for Experiments 1a and b. Long delay conditioning training occurred in Context 1 for all subjects. On Day 27, subjects received either 20 A-US pairings (Groups LongDelay.Same and LongDelay.Diff) or 20 B-US pairings (Group Control) following the same procedure described for Experiment 1a. These pairings took place in Context 1 for Group LongDelay.Same and half the subjects in Group Control, and in Context 2 for Group LongDelay.Diff and the remaining half of the Group Control subjects.

All statistical analyses were performed following the guidelines outlined for Experiments 1a and b. The data from one subject in Group LongDelay.Same was excluded from the long delay conditioning analyses due to its final segment CS A score meeting the outlier criterion. No other long delay conditioning or test score met the outlier criterion.

### Results and Discussion

**FIGURE 6:**
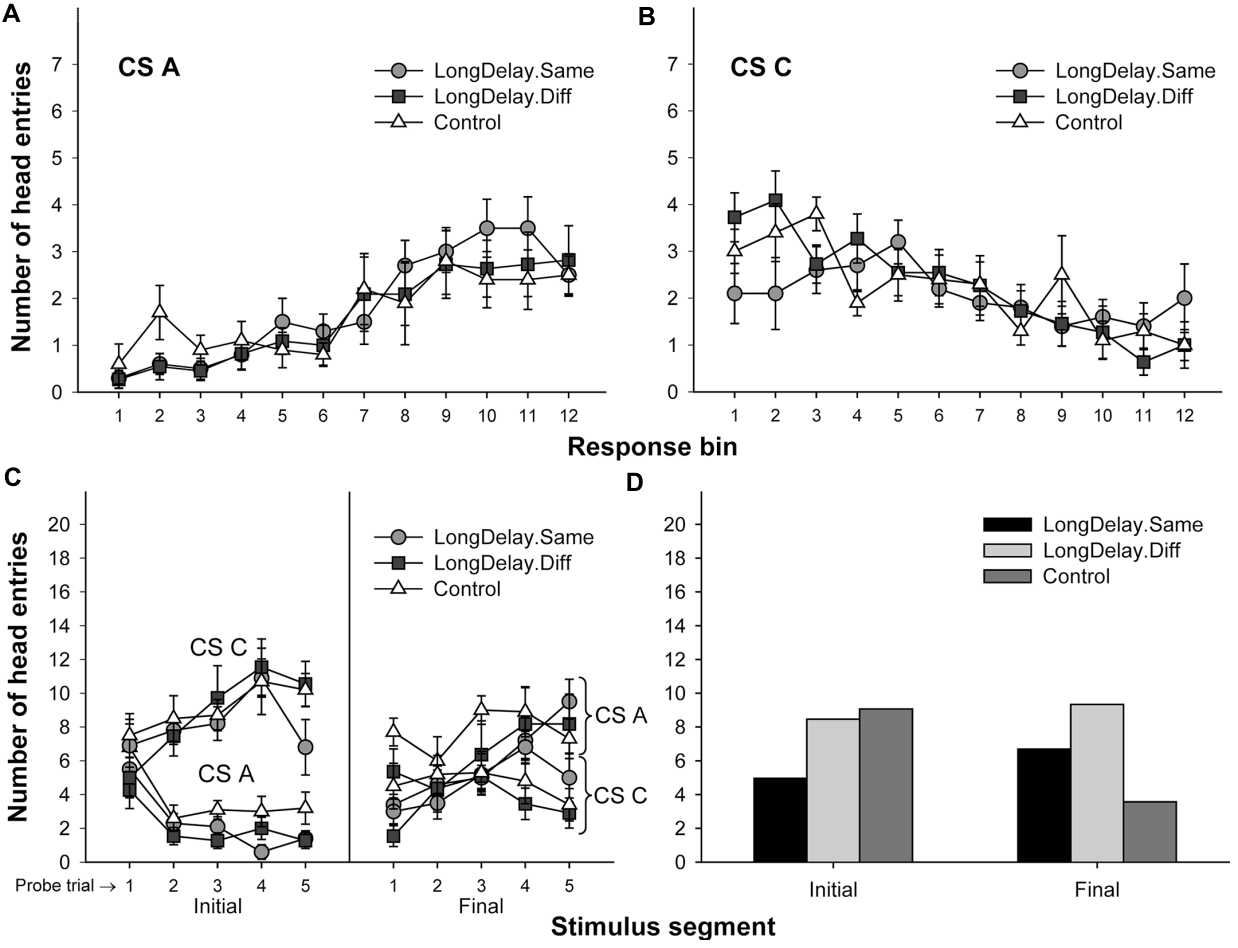
**Results of Experiment 2.** The top panels represent responding to CSs A **(A)** and C **(B)** during each of the 12 5-s bins of the last probe trial of long delay conditioning training. US delivery was expected at 55 s (Bin 11) or 10 s (Bin 2) after onset of CSs A and C, respectively. **(C)** Presents responding to the initial (average of Bins 1–3) and final (average of Bins 10–12) segments of CSs A and C during the five probe trials of long delay conditioning training. **(D)** Presents responding to the initial and final segments of CSs A (Group LongDelay) and B (Group Control) during the probe trial of retardation training, adjusted for overall rates of responding during retardation training. See text for details.

Long delay conditioning resulted in all groups exhibiting more responding to the final than the initial segment of CS A and more responding to the initial than the final segment of CS C (see **Figures 6A–C**). Groups LongDelay.Same and Control replicated the results of Experiment 1a: retardation of acquisition of a conditioned response to the initial segment of CS A was observed. Importantly, this difference was not observed when Groups LongDelay.Diff and Control were compared. That is, changing the context between long delay conditioning training and retardation training attenuated the retardation effect. The following analyses support these conclusions.

#### Long Delay Conditioning Training

A 3 (group, between groups factor) × 2 (stimulus: A vs. C, within-subjects factor) × 2 (stimulus segment: initial vs. final, within-subjects factor) ANOVA conducted on the last probe trial of long delay conditioning training revealed a Stimulus × Segment interaction, *F*(1,28) = 70.63, *MSE* = 15.20, *p* < 0.0001, and a marginally significant main effect of stimulus, *F*(1,28) = 4.13, *MSE* = 13.30, *p* = 0.052. Unexpectedly, there was also a significant interaction of Segment × Group, *F*(2,28) = 7.15, *MSE* = 7.88, *p* < 0.005. No other main effects or interactions were significant, all *p*s > 0.35. The Segment × Group interaction suggests differential acquisition across groups. To further investigate the source of this interaction and take into consideration the marginal main effect of stimulus, a series of 3 (group) ×2 (segment) ANOVAs was conducted on the data collected during the last probe trial of each individual stimulus. An analysis of CS A revealed a main effect of segment, *F*(1,28) = 49.73, *MSE* = 12.62, *p* < 0.0001, but no main effect of group nor an interaction, both *p*s > 0.21. A similar analysis of CS C revealed a main effect of segment, *F*(1,28) = 43.32, *MSE* = 10.46, *p* < 0.0001. Importantly, this analysis also revealed a Segment × Group interaction, *F*(1,28) = 4.88, *MSE* = 10.46, *p* < 0.05. The source of this interaction appears to be the relatively similar responding to the two segments of CS C in Group LongDelay.Same, which resulted in lower responding to its initial segment and higher responding to its final segment than in the other two groups (notably, the pattern of responding was the expected across trials; see **Figure 6**). Because CS C was not of relevance in the present study and responding was equivalent across groups when CS A was analyzed, we concluded that acquisition had been equivalent across groups (see **Figures 6A,C**).

#### Retardation Test

A preliminary analysis of the retardation training data revealed that the relative novelty of the context used in the Different condition adversely affected overall rates of responding. Specifically, subjects receiving retardation training in a context different from that used for long delay conditioning produced about 15% fewer responses throughout the retardation test session than subjects receiving retardation training in the same context (such differences in responding between contexts were not observed during long delay conditioning training). In consequence, retardation data were analyzed using overall rate of responding as a covariate in a 3 (group) × 2 (segment) analysis of covariance (ANCOVA), which yielded a Group × Segment interaction, *F*(2,28) = 13.89, *MSE* = 5.85, *p* < 0.0001, and marginal main effects of group, *F*(2,28) = 3.02, *MSE* = 17.83, *p* = 0.07, and segment, *F*(1,28) = 3.58, *MSE* = 5.85, *p* = 0.07. Planned comparisons revealed that responding to the initial segment of the test CS was lower in Group LongDelay.Same than Groups LongDelay.Diff and Control, *F*s(1,28) = 6.18 and 9.06, *p*s < 0.05 and 0.01, respectively. Groups Control and LongDelay.Diff did not differ, *F*(1,28) < 1. That is, acquisition of responding to the initial segment was retarded only when the context of retardation training was the same as the context of long delay conditioning. Analyses of responding during the final segment of the test CS revealed that Groups LongDelay.Diff and Control differed, while this difference was marginal for Groups LongDelay.Same and Control, *F*s(1,28) = 12.04 and 3.71, *p*s < 0.005 and 0.065, respectively. The unadjusted means for responding during the initial/final segment of the test CS were 5.36/7.27, 7.72/8.26, and 9.40/4.00, for Groups LongDelay.Same, LongDelay.Diff, and Control, respectively (least squared means, adjusted for the covariate (presented in **Figure 6D**) were 4.96/6.69, 8.40/9.33, and 9.10/3.57, respectively).

## Discussion

The term inhibition of delay has been used to describe either situations in which there is minimal or no conditioned responding to the initial segment of a long delay CS, or situations in which conditioned responding to the initial segment of a long delay CS decreases as training progresses. The former use of the term would suggest that the initial segment of the CS acquires no behavioral control, whereas the latter would suggest that the initial segment of the CS acquires behavioral control that decreases as acquisition of the temporal relationship between CS and US progresses, possibly due to inhibitory processes (cf. Pavlov, 1927). In three studies, rats were trained to retrieve food pellets delivered during the final segment of a long delay CS. Throughout training, behavior became temporally specific, with most of the conditioned responding occurring during the final segment of the long delay CS. The initial segment of the CS elicited little conditioned responding, which is the characteristic pattern that results from long delay conditioning. Despite this pattern of behavior being commonly known as *inhibition of delay*, we obtained little evidence of conditioned inhibition developing to the initial segment of the CS. The initial segment of the stimulus passed a retardation test for conditioned inhibition (Experiments 1a and 2) but failed to pass a summation test for conditioned inhibition (Experiment 1b). In Experiment 2, a context change was imposed between the long delay conditioning and retardation training phases. When the context of long delay conditioning training was different from the context of retardation training, retardation was attenuated. These three characteristics (passing a retardation test, failure to pass a summation test, and context-dependence of retardation) closely resemble the defining characteristics of the CS-preexposure effect (for a review see e.g., Escobar et al., 2003) and suggest that response attenuation in the initial segment of a long delay CS may be the result of the cumulative effects of repeated exposures to a non-reinforced stimulus segment. That is, at least in the present preparation, long delay conditioning appears to result in the development of latent inhibition, rather than ‘true’ conditioned inhibition, to the initial segment of the CS.

Our observation that the initial segment of the long delay CS failed to pass a summation test in Experiment 1b should not be viewed as definitive evidence against the possibility that conditioned inhibition develops to the initial segment of a long delay CS for several reasons. First, some authors have regarded passing of a retardation test as sufficient evidence of conditioned inhibition, as long as the alternative explanation of attenuated attention to the inhibitor can be precluded with appropriate control conditions (Papini and Bitterman, 1993). Furthermore, other authors have claimed that passing just a summation or a retardation test should be considered sufficient evidence of inhibition because multiple variables (e.g., collateral excitation) may preclude a stimulus from passing both tests (Williams et al., 1992). However, passing both tests is still regarded by most researchers as necessary to conclude that a treatment is conducive to the development of conditioned inhibition (e.g., Cole et al., 1997), and this two-test strategy (cf. Rescorla, 1969) is frequently used as a behavioral definition of inhibition (Savastano et al., 1999).

Previous studies on inhibition of delay have reported evidence of inhibition as assessed with a summation test. For example, Rescorla (1967) trained dogs to fear a 30-s tone that coterminated with a 5-s shock. This tone was later used as a warning signal in a discriminated avoidance preparation. The long delay conditioning treatment resulted in the initial 5 s of the CS attenuating fear to the training context; that is, the number of avoidance responses during the initial segment of the CS fell below baseline level. Thus, one can conclude that the long delay CS passed a summation test for conditioned inhibition. We did not observe a similar attenuation: mean number of head entries during the period of time immediately preceding CS onset was 1.5 and during the initial segment of the CS was 2.25 (these numbers were taken from the last probe trial data across studies, using 15 s as window for both measures). This discrepancy may be due to conditioned inhibition developing at different rates in aversive (Rescorla’s) vs. appetitive (Pavlov, 1927; the present studies) conditioning. Because conditioned inhibition appears to be a positive function of the number of training trials administered (e.g., Heth, 1976; Yin et al., 1994; Cole and Miller, 1999; Stout et al., 2004), further increasing the number of long delay conditioning trials might favor the development of conditioned inhibition in the present preparation. Notably, in a preparation similar to that used in the present experiments, Williams et al. (2008b) observed attenuated responding to the initial segment of a long delay CS, as compared to the preceding ITI. In Williams et al.’s (2008b) preparation, the CS-US contingency was degraded by delivering unsignaled pellet USs during the intertrial intervals; thus, this response attenuation may have been due to the degraded contingency rather than (or in addition to) the long delay conditioning training.

It is possible that the development of robust inhibition in the long delay procedure requires that the training excitor be highly excitatory. A problem with this latter possibility is to determine what stimulus acts as the training excitor for the initial segment of the long delay CS. A likely candidate is the final segment of the CS, which is contiguous with the US. However, conditioned inhibition is most readily obtained when the training excitor is presented in a non-reinforced simultaneous compound with the candidate inhibitor. Although there are some reports of inhibition when the training excitor and the putative inhibitor are presented serially (e.g., Rescorla, 1985; Stout et al., 2004), this type of inhibition generally develops more slowly, requiring many more compound trials than the simultaneous type of inhibitory training (Stout et al., 2004) and sometimes fails to result in inhibition at all (e.g., Holland, 1985). Because the initial and final segments of a long delay CS are separated by time, neither compound training nor close proximity training can take place. A second candidate excitor is the training context. During long delay conditioning training, the context is paired *both* with the US and the initial segment of the CS. Assuming that the context plays the role of training excitor can explain some of our results as well as some of the apparent discrepancies between the previous literature and the present studies. For example, because of the relatively extensive exposure to the training context in our preparation, the context may not have been excitatory enough to support robust inhibition. During each of the 25 training sessions, the context was paired with the US approximately 20 times (18 times during the 5 probe days, 20 times the remaining 20 days) and was presented in the absence of the US for about 118 min in each of these 25 training sessions. This relatively massive context extinction should have attenuated the impact of the context-US pairings, thus resulting in low excitation to the context (see Gibbon and Balsam, 1981; Gallistel and Gibbon, 2000, for a description of how long durations of exposure to the context without the US might attenuate contextual excitation). In Rescorla’s study, both the final segment of the CS and the training context could have been more effective as training excitors because, in his study, the CS duration was 30 s, which is half of the duration of the CSs in the present studies. Thus, it is more likely that the excitatory properties of the final segment of the CS affected learning about the initial segment of the CS (closer temporal proximity), and the significantly shorter non-reinforced exposure to the context made it more excitatory than in the present studies (but see Detert et al., 2008, for evidence that rats acquire little fear of the context during long-delay conditioning training).

It has been long known that animals can delay responding until the expected time of US delivery. Furthermore, recent reports suggest that animals also encode the specific time of US omission (e.g., Denniston et al., 1998a,b; Burger et al., 2001; Williams et al., 2008a). The present results support the view that time is a relevant variable both for expectation of US delivery and expectation of US omission. Furthermore, they support the view that different segments of a CS may acquire different associative meanings (i.e., excitatory and inhibitory response potentials). Consistent with this view, Romaniuk and Williams (2000) observed that backward conditioning results in excitation to the CS segment that is contiguous with US delivery, even if the CS as a whole was inhibitory. Similarly, Williams et al. (2008a) observed that conditioned inhibition is specific to the expected time of US omission, which is determined by the time of reinforcement of the training excitor. Taken together, these results indicate that information about US delivery and US omission can coexist through the duration of a CS, and development of excitation and inhibition, respectively, is determined by the temporal information encoded as part of the association. Our results add to this body of research by demonstrating that a segment of a CS that *predicts* the future delivery of the US can signal a relationship with the US different from excitation.

The present experiments suggest that a stimulus (in the present case, a long delay CS) should not be viewed as a unitary event but as a sequence of events that occurs over time. The initial segment of the CS has distinct properties, including the change in stimulation that accompanies stimulus onset. Similarly, the final segment of the CS has distinct properties, including CS duration and termination. Real-time associative learning theories should be well suited to account for this perspective and the present results, especially if they assume that CS perception is achieved through some sort of stimulus sampling process (cf. Estes and Burke, 1953). For example, recent extensions of Wagner’s (1981) SOP model (e.g., C-SOP; Wagner and Brandon, 2001) propose combining a componential representation view with a competitive learning rule to account for the response pattern characteristic of inhibition of delay. According to Vogel et al. (2003; also see Brandon et al., 2003), the sampling of CS components is not random, but determined by a temporal process, such that CS components with greater proximity to the US become more strongly associated to the US than others (Wagner’s original model assumed random sampling). When the componential representation and competitive learning views are combined, the model predicts the pattern of responding characteristic of inhibition of delay. Briefly, C-SOP assumes that delayed responding in long delay conditioning should be viewed as an instance of an AX+, BX- discrimination, where A, B, and X represent CS components that are differentially activated due to their association with the US. A-elements are those uniquely activated during US presence, B-elements are those uniquely activated during US absence, and X-elements are those activated during either CS presence or CS absence. In consequence, A-elements are assumed to become strongly excitatory, X-elements moderately excitatory, and B-elements just inhibitory enough to counter the excitation from X-elements during BX joint activation.

The C-SOP approach appears consistent with our data because it represents long-delay conditioned stimuli as neutral, rather than inhibitory, in their initial stages (BX elements) and excitatory in their final stages (AX elements). However, the model would need to account for *both* retarded acquisition of responding to the (presumably neutral) initial segment of the CS, and the context-specificity of the effect observed in Experiment 2. Retardation could be addressed by assuming that the B elements are inhibitory enough that their associative strength would grow at a slower rate after long delay conditioning than for a novel stimulus. Context specificity could be addressed by assuming that the B elements become strongly associated to the context, which would lead to the prediction of latent inhibition, a phenomenon explained by SOP in terms of strong CS-context association making some CS elements unavailable to later enter into associations with the US (Wagner, 1981). However, latent inhibition should be weak in this situation because at least some of the B elements would be inhibitory and unavailable to enter into associations with the context.

There are potential alternative explanations for our data. For example, it is possible that the initial segment of the long delay CS underwent habituation, which could have in turn resulted in retardation (a habituated stimulus segment may be less ready to enter into associations with the US when reinforced) and a failure of summation (a habituated stimulus would detract little responding from the transfer excitor). Long-term habituation and latent inhibition evolve from similar operations (see Lubow, 1989, for an extensive discussion), and some theoretical frameworks suggest that the two may be the result of a common underlying process (e.g., Wagner, 1976). The present studies do not allow for a full dissociation of latent inhibition and long-term habituation, and further research would be needed to obtain evidence for such a dissociation. However, Experiment 2 may provide greater support to the hypothesis that latent inhibition rather than habituation developed to the initial segment of the long delay CS because, at least under some circumstances, latent inhibition exhibits greater context dependence than habituation (see e.g., Hall, 1991). Another possible explanation for our data is that the initial segment of the CS develops weak associations to later segments of the CS or the US due to the relatively long duration of the inter-stimulus interval (ISI, defined as the interval between CS onset and US onset). However, duration of the ISI alone does not account for the development of long delay conditioning. For example, situations in which the ISI is maintained constant but the CS is of short duration (i.e., if a trace is introduced between CS termination and US onset) result in similar levels of responding and develop at similar ontogenetic times as long delay conditioning (Barnet and Hunt, 2005). However, trace conditioning and long delay conditioning appear to be mediated by different physiological systems. Trace conditioning is disrupted by the cholinergic antagonist scopolamine and enhanced by the cholinesterase inhibitor physostigmine, neither of which has an effect on long delay conditioning (Hunt and Richardson, 2007). These observations can be viewed as problematic for our conclusions, considering that latent inhibition is disrupted by low doses (<0.5 mg/kg) of scopolamine (Barak and Weiner, 2007). Nonetheless, the reports that scopolamine spares long delay conditioning used a higher dose of scopolamine (1.0 mg/kg), and at high doses scopolamine does not disrupt but actually enhances latent inhibition (Barak and Weiner, 2009). These latter observations are consistent with our conclusion that long delay conditioning results in at least some degree of latent inhibition, likely mediated by the development of strong associations between the CS and the context during long delay exposure to the CS (cf. Escobar et al., 2002).

The present data suggest that stimuli should not be viewed as unitary events, but as a sequence of temporally linked events that can carry different types of information about the conditions that precede and follow them. The use of associative strength as a singular summary statistic for the associative value of a stimulus may be an oversimplification that ignores that different segments of the CS may carry different associative meanings with respect to US delivery and US omission.

## Conflict of Interest Statement

The authors declare that the research was conducted in the absence of any commercial or financial relationships that could be construed as a potential conflict of interest.
